# A Soft Exoskeleton for Hand Grip Augmentation and Fall Prevention Assistance in Tower Climbing

**DOI:** 10.3390/biomimetics10110721

**Published:** 2025-10-29

**Authors:** Shaojian Fu, Zuyuan Chen, Lu Gan, Jingqi Ling, Hao Huang, Junkai Chen, Yitong Zhou

**Affiliations:** 1 The Shien-Ming Wu School of Intelligent Engineering, South China University of Technology, Guangzhou 510641, China; 202421061224@mail.scut.edu.cn (S.F.); 202221059937@mail.scut.edu.cn (Z.C.); wiganlu@mail.scut.edu.cn (L.G.); 202321060324@mail.scut.edu.cn (H.H.); wijunkaichen@mail.scut.edu.cn (J.C.); 2 Department of Fashion and Accessories Design, Guangzhou Academy of Fine Arts, Guangzhou 510006, China; jingqi@gzarts.edu.cn

**Keywords:** soft exoskeleton, energy storage–release mechanism, grip augmentation, fall prevention assistance

## Abstract

This study presents a soft exoskeleton system designed to enhance the safety of electrical maintenance personnel during tower climbing by augmenting the hand grip and providing fall prevention assistance. Inspired by biological principles, a compact, stroke-amplified, and fast-response actuator based on a spring energy storage–release mechanism was developed and evaluated through tensile and speed tests, demonstrating sufficient locking force and a fast response time of 37.5 ms. A dual-sensing module integrating pressure and flexible bending sensors was designed to detect grasping states in real time. System effectiveness was further validated through functional electrical stimulation (FES) and simulated climbing experiments. FES tests confirmed the system’s ability to maintain grasp posture under involuntary hand extension, while climbing experiments verified consistent and reliable transitions between locking and unlocking during movement. Although preliminary, these results suggest that integrating soft exoskeletons with rapid-response actuators offers a promising solution for improving grip stability and operational safety in high-risk vertical environments.

## 1. Introduction

Electric power maintenance operations still heavily rely on manual labor [1], with workers needing to climb for extended periods to perform inspection and maintenance on high-voltage power towers [2,3]. Due to the height and environmental complexity of power tower climbing operations, workers face a significant risk of falling. In 2017, Poland reported a total of 5379 high-altitude fall accidents [4]; in the United States, high-altitude falls accounted for one-quarter of all reported workplace injury incidents in 2019 [5]. These statistics indicate that frontline workers are at high risk of falling from heights. As a result, effectively preventing fall accidents and ensuring worker safety have become critical issues that the power industry urgently needs to address.

Currently, workers involved in high-altitude operations primarily rely on personal fall protection equipment, which can be categorized into three types based on their function [6]: positioning systems for maintaining work posture during high-altitude operations; fall arrest systems for stopping a fall that has already occurred [7,8]; and fall prevention systems for preventing the initial occurrence of a fall [9].

Among these, positioning systems for high-altitude operations are suitable for suspended work but are not applicable to tower climbing. Common fall protection devices used on transmission towers include foot peg-style, track-based, and steel cable fall arrest systems. Foot peg devices require both hands to operate fall arrest hooks, making the process cumbersome, which often leads workers to avoid using them despite safety regulations mandating their use [10], while track-based and steel cable systems, although requiring no manual operation, suffer from high installation costs, limiting their practical application [10,11,12]. These devices are all part of fall arrest systems, which can only mitigate impact through braking mechanisms (such as safety harnesses or rails) once a fall has occurred and cannot actively help prevent falls [13]. Furthermore, they generally have a large mass and require modifications to the tower structure, limiting their widespread use. Therefore, there is an urgent need for a new type of fall prevention solution that can prevent falls, does not require tower structure modifications, and can operate conveniently.

Exoskeleton technology, with its flexibility and ability to assist human movements, offers new insights into addressing safety issues in complex climbing environments [14,15]. Currently, exoskeletons driven by gas [16,17], shape memory alloys [18], and motors [19,20,21,22] have been widely applied in the fields of assistive technology [23,24,25,26] and rehabilitation [27,28,29,30]. For the specific scenario of climbing power towers, previous research has made preliminary advancements in developing specialized exoskeleton systems for hand grip assistance [31] and hip joint movement assistance [32]. However, this scenario presents a dual challenge to system performance. First, skilled workers climb at speeds of 45 to 67 steps per minute (with a frequency of approximately 0.7 to 1.1 Hz) [33], while existing exoskeletons struggle to match this response speed [34,35,36,37]. Second, traditional exoskeletons often lack transparency during the driving process, imposing additional forces on the body and interfering with the user’s movements, which affects climbing efficiency and safety. Therefore, developing an exoskeleton system that responds quickly, offers high transparency, and adapts to high-frequency climbing motions remains a significant challenge.

To better position this study within recent advances in climbing- and safety-oriented exoskeletons, a comparative analysis was added (Table 1). Table 1 summarizes representative works published between 2023 and 2025 that focus on soft or hybrid exoskeletons for grip enhancement, tower climbing, and industrial safety. Existing studies mostly emphasize passive or post-event assistance, such as magnetorheological grip reinforcement [38] or metabolic optimization during stair ascent [39], but none address proactive fall prevention in high-altitude operations. In contrast, our system integrates a spring-based rapid-locking actuator and a dual-sensor fusion grip recognition algorithm, enabling predictive locking before slip occurs. Its 37.5 ms response is faster than the <50 ms mechanical grasping reported by Roderick et al. [40] and better suited to human climbing cadence (1 Hz). Additionally, the glove’s modular two-finger constraint and low-friction PTFE tubes improve comfort and minimize cable interference, aligning with the ergonomic surface adaptation trend of Mahuttanatan et al. [41]. Together, these features shift the functional focus of climbing exoskeletons from passive assistance to active safety assurance.

In nature, certain creatures have evolved highly efficient actuation strategies by leveraging elastic energy storage–release mechanisms. These strategies involve preloading elastic structures, such as leaves or muscles [42], with prestress to store potential energy that can be rapidly released, enabling swift and powerful movements. This approach yields fast response, high force output, and notable energy efficiency. For example, the Venus flytrap [43] captures prey through the rapid release of elastic energy stored in its bistable leaves, while reptiles and amphibians, such as chameleons [44], use similar mechanisms in their tongues to catch insects with remarkable speed. A number of bioinspired robots have demonstrated the effectiveness of using such strategies for achieving high-speed and payload actuation [45,46,47,48]. Inspired by these biological strategies, this study adopts a spring-based energy storage–release mechanism to realize rapid hand locking within a compact actuator. Building on this actuator design, a soft exoskeleton system is developed to assist tower climbers by fast switching between the locked and unlocked state of the hand, ensuring that at least one hand remains reliably engaged with a foot peg throughout the climbing process. By augmenting grip and enabling rapid locking, the system actively contributes to fall prevention assistance in high-risk vertical tasks. The main contributions are as follows:

1. A compact, stroke-amplified, and fast-response actuator based on a spring energy storage–release mechanism was developed, achieving a locking response time of 37.5 ms—among the fastest reported for exoskeletons. The actuator exhibits high transparency and does not impede natural hand movement during non-locking phases.
2. A dual-sensor fusion module integrating pressure and flexible bending sensors was proposed to reliably detect the user’s handgrip condition in real time, ensuring accurate and robust triggering during climbing activities.
3. A soft wearable exoskeleton system was developed and integrated, and its effectiveness in augmenting grip and climbing safety was experimentally validated through functional electrical FES tests and simulated tower climbing tests.

**Table 1:** Recent developments in exoskeleton technology.

Reference (Year)	Exoskeleton Type	Actuation/Control	Response Time	Weight	Wearability and Application Focus
This Study	Upper-limb tower climbing exoskeleton	Spring + servo motor	37.5 ms	1.96 kg	Velcro + wrist anchor; wrist–tower non-interference
Li et al., 2024 [32]	Climbing hip exoskeleton	GO-M8010 motor + passive joints	Not specified	3 kg	Load transfer and fatigue relief
Li et al., 2025 [38]	Magnetorheological hand exoskeleton	MRG clutch + coil arrays	ms-scale	—	Velcro-based, passive grip enhancement
Park et al., 2025 [39]	Human-in-the-loop hip exoskeleton	AK80-9 motor + HIL control	200 Hz	3.6 kg	Metabolic optimization in stair ascent
Mai et al., 2025 [49]	Non-motorized hand exoskeleton	MR actuators (no external power)	Fast lock	1.7 kg	Rescue grip aid, passive locking
Mahuttanatan et al., 2025 [41]	S-DOT soft anti-slip band	Passive silicone dots	—	Lightweight (<0.2 kg)	Improves skin–surface conformity
Moya-Esteban et al., 2025 [50]	Soft back exosuit	Cable + AC servo + 24 V battery	≈27 ms delay	—	Back support, neuro-mechanical modeling

The remainder of this paper is organized as follows. Section 2 details the mechanical design and bio-inspired actuation principle of the proposed soft exoskeleton for tower climbing safety. Section 3 presents the experimental evaluation of the system’s performance, including response time, torque output, and grip recognition accuracy. Section 4 discusses the results in comparison with recent industrial and safety exoskeletons, highlights the trade-offs between comfort and force transmission, and outlines the study’s limitations and future research directions toward adaptive and field-ready implementations.

## 2. Development of the Assistive Fall Prevention Exoskeleton System

### 2.1. Design of the Assistive Fall Prevention Exoskeleton System

**Figure 1:**
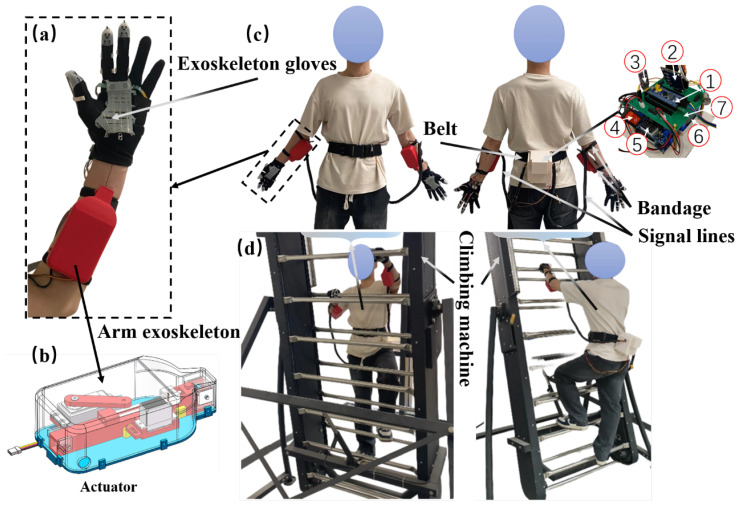
Overview of the assistive fall prevention exoskeleton system for tower climbing. (**a**) Single-arm exoskeleton. (**b**) Perspective view of the actuator structure. (**c**) Schematic diagram of the overall system worn by the user. Components of the control box: 1. STM32; 2. linear voltage conversion module; 3. Bluetooth module; 4. system main switch; 5. step-down module; 6. battery; 7. control board. (**d**) Simulating the actual climbing scenario using a climbing machine.

Figure 1 shows the overall design of the assistive fall prevention soft exoskeleton system for tower climbing. The system consists of a control box, an actuator module, an exoskeleton glove, and a dual-sensing module. These components work together to achieve the fall prevention function. The control box is secured to the user’s waist with an adjustable length belt and is responsible for coordinating the operation logic and data processing of all modules. The control box communicates with the actuator module and the exoskeleton glove through signal lines for real-time data transmission, ensuring efficient operation of the system. Figure 1a shows the exoskeleton worn on one arm, with the exoskeleton glove worn on the hand and the actuator module fixed to the forearm using an adjustable strap. The glove integrates a dual-sensing module and transmission accessories. The sensing module is used to monitor the hand’s gripping status in real time, transmitting the collected data to the control box for processing. The transmission part is detailed in Section 2.3. The internal structure of the actuator is shown in Figure 1b. Figure 1d demonstrates the user climbing a climbing machine while wearing the exoskeleton system. The system is designed to be fully portable and untethered, with a total weight of 1.96 kg.

### 2.2. Actuator Based on Spring Energy Storage–Release Mechanism

**Figure 2:**
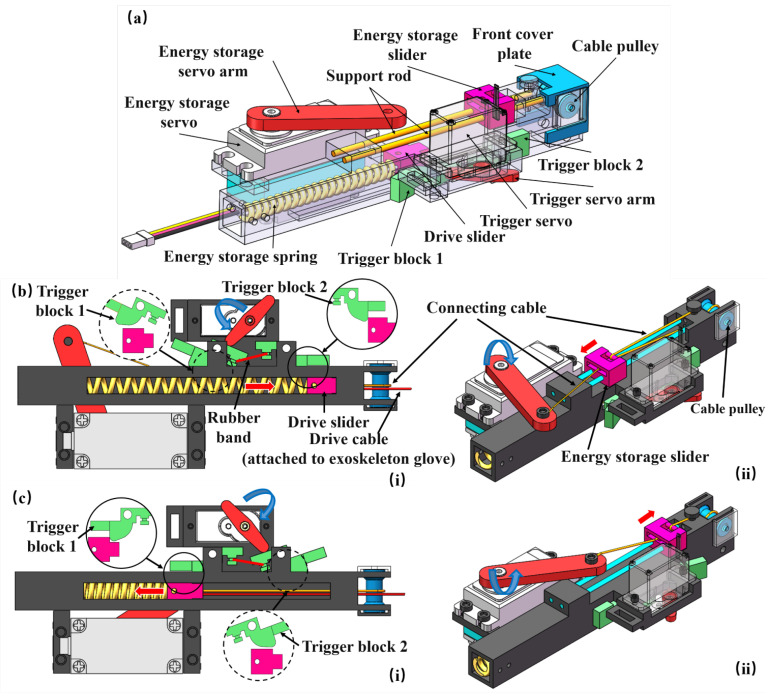
(**a**) A 3D rendering of the actuator based on a spring energy storage and release mechanism. (**b**) The actuator in the energy storage phase. (**c**) The actuator in the energy release phase. (**i**) and (**ii**) indicate different views.

As shown in Figure 2a, a compact and efficient actuator is proposed, which utilizes a spring-based energy storage and release mechanism to rapidly convert elastic potential energy into linear motion, pulling the drive cables to achieve fast hand locking in coordination with the exoskeleton glove. The energy storage and release process is coordinated by the energy storage servo and the trigger servo. Three cables are involved in transmission: one connects the energy storage servo arm to the energy storage slider; the second cable links the energy storage slider to the drive slider; and the third is the drive cable connecting the drive slider to the exoskeleton glove. To maximize spring extension within a limited servo stroke, a stroke-amplifying pulley mechanism was introduced along the second connecting cable. This mechanism consists of a movable pulley (shown as a rod) on the energy storage slider, a fixed point on the actuator frame (shown as a pin), and a fixed cable pulley on the actuator frame, forming a compound pulley system. As the energy storage slider moves a distance, *D*, the drive slider connected to the spring travels approximately 2D, effectively doubling the spring tension without increasing energy storage servo travel. This simple yet effective arrangement functions as a variable-stroke-ratio mechanism, enabling a compact and efficient design. The first two connecting cables are drawn in yellow and the drive cable in red, as shown in Figure 2b,c. The drive slider operates between two stable positions defined by the geometric constraints of trigger blocks 1 and 2. The dashed circles indicate the initial state of the trigger block mechanism, while the solid circles represent the final state. A pre-tensioned rubber band connects the two trigger blocks, enabling passive reset after the trigger servo completes its action.

As shown in Figure 2b, during the energy storage phase, the trigger servo rotates clockwise to pivot trigger block 1, removing the shape constraint on the drive slider and allowing it to move freely along the sliding channel. The energy storage servo then rotates clockwise, and its arm pulls the energy storage slider leftward via a connecting cable. The connecting cable involving stroke-amplifying pulley mechanism connects the energy storage slider to the drive slider, causing the drive slider to move rightward in the opposite direction. Once the drive slider passes trigger block 2, it is mechanically constrained. At this point, the drive slider reaches its rightmost position, closest to the glove side, and the drive cable connecting the drive slider and glove remains slack due to its fixed length, ensuring no force is transmitted to the exoskeleton glove. The spring is fully stretched and remains tensioned without external force, as its position is secured by the constraint between trigger block 2 and the drive slider. The energy storage servo then rotates counterclockwise to return to its initial position, preparing for the next cycle; during this time, the cable between the energy storage servo and the energy storage slider becomes slack. By this time, the energy storage phase is complete.

As shown in Figure 2c, during the energy release phase, the trigger servo pivots trigger block 2, removing the constraint on the drive slider. The stored spring energy is instantly released, driving the slider leftward at high speed. Once the drive slider passes trigger block 1, it is mechanically constrained again. During this rapid motion, the drive cable is pulled to its leftmost position—farthest from the glove—thereby tensioning the exoskeleton glove and locking the hand into a gripping posture. The energy storage slider is pulled back to its initial position by the drive slider via the connecting cable. By this time, the energy release phase is complete. This triggering mechanism enables fast actuation and ease of control, effectively meeting the need for rapid grip locking during climbing tasks.

**Figure 3:**
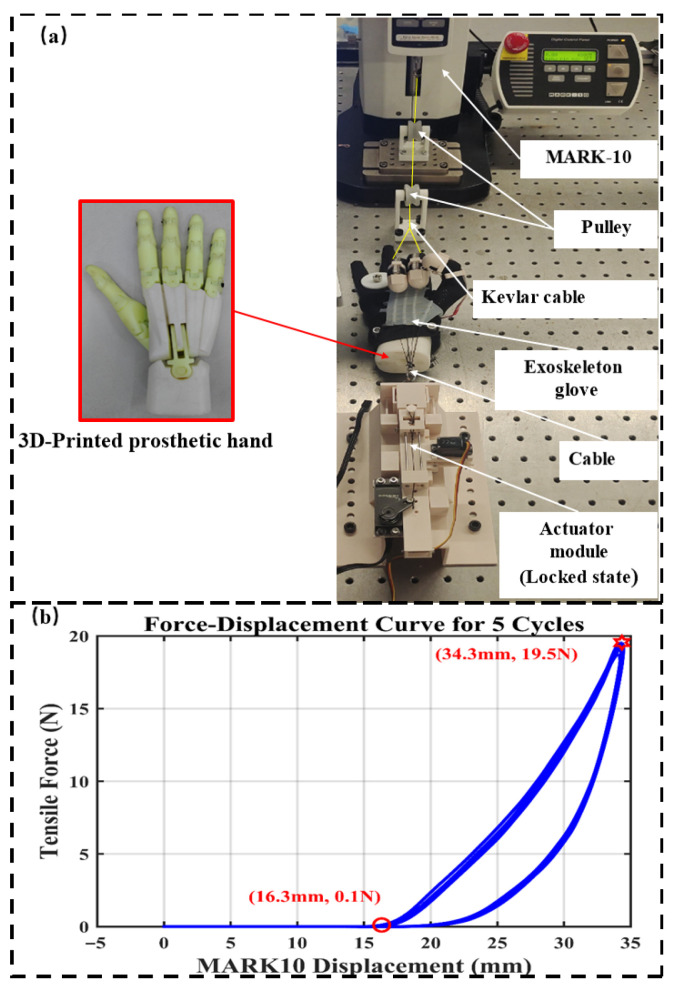
The tensile test performed to evaluate actuator locking performance. The actuator is held in the locked state with the drive slider constrained by trigger block 1. A Mark-10 tensile machine applies tensile force to the proximal interphalangeal joints of the index and middle fingers on a prosthetic hand wearing the exoskeleton glove. (**a**) The experimental setup. (**b**) The measured tensile force and corresponding displacement.

To evaluate the locking performance of the actuator, a tensile test was conducted on the actuator in its locked state, as shown in Figure 3a. To ensure that the measured locking force originates solely from the fast actuator, a soft exoskeleton glove was worn on a 3D-printed prosthetic hand. During testing, the fast actuator and the soft exoskeleton glove were maintained in a locked state. A tensile testing machine (MARK-10 ESM303, MARK-10 CORPORATION, New York, NY, USA) was used to apply a tensile load to the proximal interphalangeal joints of the index and middle fingers of the model hand. The maximum tensile force applied in the test was set to 20 N, and the experimental results are presented as a force–displacement curve in Figure 3b. The results indicate that when the exoskeleton system is subjected to a 20 N tensile force at the proximal interphalangeal joints of the index and middle fingers, the corresponding horizontal displacement of the hand exoskeleton is 15.6 mm. A preliminary qualitative assessment was performed by placing a 0.2 kg weight (2 N) on the middle joint of the index finger during a gripping posture. While limited in scope and subjective in nature, the participant reported noticeable resistance when attempting finger extension, suggesting that even moderate opposing force may significantly hinder extension. This supports the assumption that a locking force of approximately 20 N (i.e., 10 N per finger) is sufficient to stabilize the hand grip against fatigue-induced or involuntary release during climbing. This displacement, when assigned to the movement of the finger joint, corresponds to a rotation angle of approximately 9°. These findings demonstrate that the actuator allows only minimal finger bending under substantial external tensile force, thereby ensuring effective locking performance. Although the mechanical constraint between trigger block 1 and the drive slider is secure, displacement still occurs under the tensile force applied by the testing machine. This displacement is primarily attributed to deformation of the glove, relative sliding between the glove and the hand, and motion between components such as the C-shaped rings and the glove itself—common phenomena in wearable soft exoskeletons. As expected, when worn by a human, additional deformation may arise due to the inherent compliance of the hand. To mitigate such deformation, several strategies can be employed: improving the glove’s fit by selecting a smaller size to achieve tighter contact with the hand; replacing 3D-printed accessories with metal components to enhance structural rigidity; or increasing constraint along the transmission path, such as reducing the inner diameter of the C-shaped rings. However, some strategies, such as the latter, may compromise the comfort of wearing. Therefore, in the design, a trade-off between comfort and transmission efficiency should be carefully considered.

**Figure 4:**
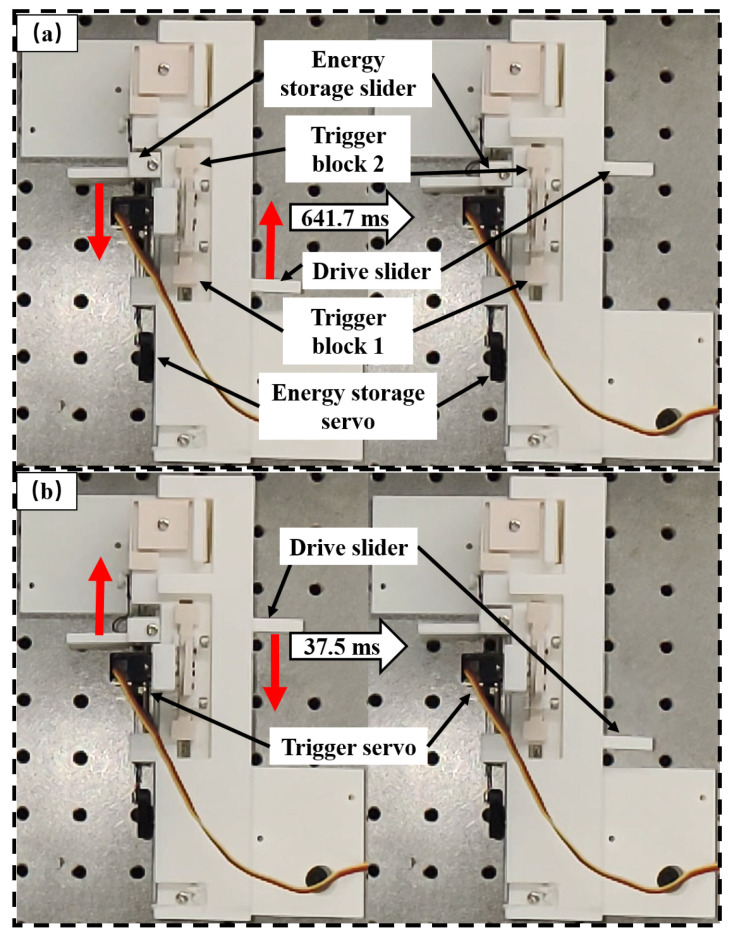
Actuator speed test. (**a**) Energy storage phase: The energy storage servo rotates, pulling the energy storage slider downward and the drive slider upward. Once the drive slider is constrained by trigger block 2, the energy storage servo and slider return to their initial positions. (**b**) Energy release phase: The trigger servo pivots to release trigger block 2, allowing the spring to rapidly pull the drive slider downward until it is constrained by trigger block 1.

To evaluate the speed of the fast actuator, a high-frame-rate camera operating at 240 frames per second was used to capture its operation. Frame-by-frame analysis was conducted to quantify the duration of each phase. For better visualization of internal motion, the energy storage slider and drive slider were slightly modified without affecting the actuator’s dynamic response. The energy storage phase, as defined in Section 2.2, refers to the time interval between the initiation of the energy storage servo movement and the return of the energy storage servo to its initial position. This duration was measured to be 641.7 ms, as shown in Figure 4a. The energy release phase, also defined in Section 2.2, corresponds to the time between the initiation of the trigger servo and the moment the drive slider is constrained by trigger block 1. The measured duration of the energy release phase was 37.5 ms, as shown in Figure 4b. Combining the two phases, the total duration of one complete actuation cycle is 679.2 ms, corresponding to an actuation frequency of approximately 1.5 Hz, which satisfies the required actuation frequency in climbing scenarios [33].

### 2.3. Sensing Module Design and Calibration

**Figure 5:**
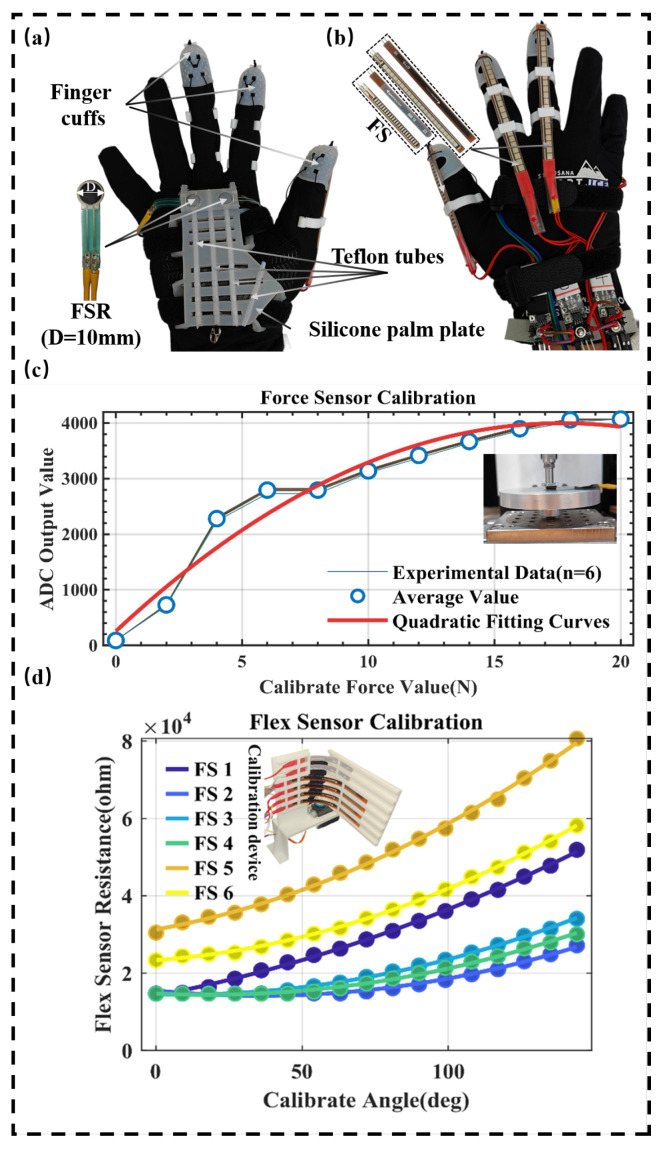
Exoskeleton glove overview and sensor calibration. (**a**) Front view of the exoskeleton glove. (**b**) Back view of the exoskeleton glove. (**c**) Thin-film pressure sensor calibration results. (**d**) Flexible bending sensor calibration results.

To ensure reliable grip recognition and prevent unintended activation, a dual-sensing strategy combining finger bending and palm pressure sensors was implemented. The sensing module comprises three flexible bending sensors (Spectra Symbol, Salt Lake City, UT, USA) and two thin-film pressure sensors(FSR, RP-C10-LT, LEGACT Technology, Shenzhen, China), as illustrated in Figure 5a,b. They are used to measure palm contact and the bending status of the middle finger, index finger, and thumb. This design leverages the complementary characteristics of the two sensing modalities: finger flexion alone, such as forming an empty fist, activates only the bending sensors but not the pressure sensors; conversely, pressing the palm against an object without actual grasping may activate the pressure sensors but not the bending sensors. By requiring simultaneous detection from both channels, the system robustly distinguishes genuine gripping actions from incidental hand movements, thereby significantly reducing false triggers.

The FSR sensors were calibrated using a tensile testing machine (MARK-10 ESM303, USA). During the calibration process, the resistance of the FSR sensors was measured under varying pressure conditions. The data were fitted using a quadratic function, as illustrated in Figure 5c. The bending sensors were calibrated using a servo-driven platform that bent each sensor to specified angles. The data were fitted using a quadratic function. The calibration results for both sensor types demonstrated a goodness of fit ($R^{2}$) exceeding 0.99 across all six sensors. The calibration device and results are shown in Figure 5d.

### 2.4. Exoskeleton Glove Design

The exoskeleton glove consists of finger sleeves, finger rings, drive cables, silicone palm plates, Teflon tubes, and straps, as shown in Figure 5a. The drive cable is a polyethylene (PE) wire, with a diameter of 0.55 mm. The finger sleeves are made of silicone material, offering good flexibility and stretchability to ensure comfort and a snug fit. The finger rings are 3D-printed using polylactic acid (PLA) and feature a “C”-shaped semi-open ring structure that enhances adaptability to different finger sizes. Each finger ring has two circular channels at one end, which serve as anchor points for the drive cables; the other end has a rectangular channel to secure the flexible bending sensors. This design not only ensures stable positioning of the cables and sensors but also improves the system’s reliability and operational precision.

The silicone palm plate, serving as the core support structure of the glove, is produced using a casting process and incorporates thin-film pressure sensors and Teflon tubes embedded inside. Teflon tubes, being low-friction plastic pipes, are used as conduits for the drive cables, ensuring smooth transmission during cable movement while also protecting the cables.

**Figure 6:**
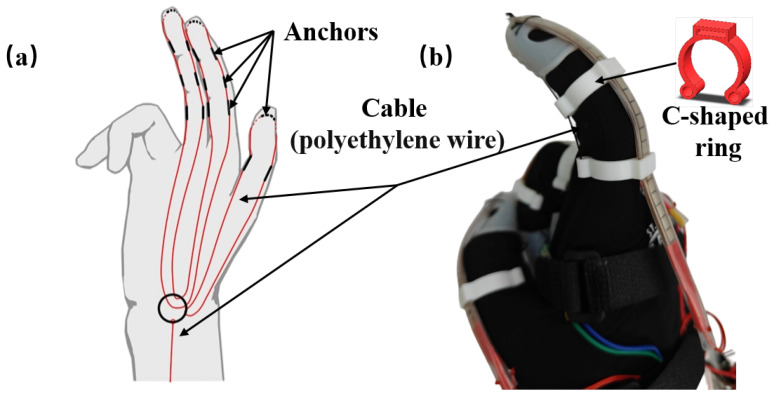
The cable routing scheme for the fingers. (**a**) A schematic of the drive cable routing. (**b**) A single-finger display of the exoskeleton glove.

Figure 6a,b show the detailed cable routing layout. A differential wiring scheme [19] is adopted to reduce the number of actuators and overall system complexity while still ensuring balanced force distribution across the thumb, index, and middle fingers. To facilitate effective force transmission and minimize deformation of the wearable structure, three sets of anchor points are arranged along each finger. The distal anchor point is located on the dorsal side of the fingertip (near the nail) and is directly provided by the finger cuff, whereas the middle and proximal anchor points are realized through rings fixed onto the glove and connected to the finger sleeves.

## 3. System Performance Experiments

To verify the performance and effectiveness of the proposed fall prevention soft exoskeleton for tower climbing, a comprehensive evaluation was conducted, including functional electrical stimulation tests and climbing machine experiments.

For this prototype verification phase, subject recruitment was limited by equipment and time, with only one 24-year-old healthy male subject (170 cm, 65 kg, dominant right hand, and no upper limb movement disorders) was included. In the FES experiment and climbing machine experiments, the subject completed five independent trials (each climbing task was repeated five times at 1 Hz, close to the real tower climbing frequency) with ≥5-min intervals between trials to avoid short-term fatigue. All experiments were conducted under controlled laboratory conditions, with the climbing machine fixed at 72° (approximating real power tower angles) to reduce environmental confounding effects.

### 3.1. Functional Electrical Stimulation Experiments

To simulate unintentional hand opening caused by fatigue or inattention during climbing, an FES experiment was conducted. FES delivers electrical impulses to motor nerves to induce involuntary muscle contractions and is widely used in rehabilitation and medical applications. This experiment aims to evaluate the locking effectiveness of the proposed soft exoskeleton system under such involuntary hand opening conditions [51].

**Figure 7:**
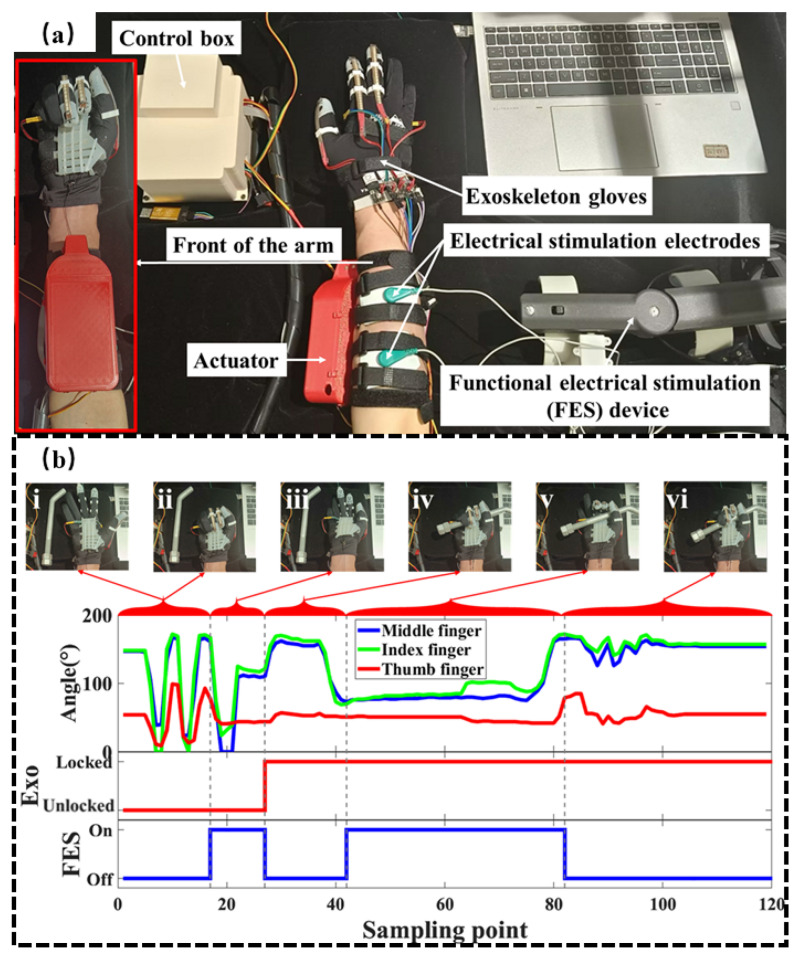
FES test (**a**) setup and (**b**) results. Angle: right-hand flexion angle, Exo: exoskeleton.

The experimental setup is illustrated in Figure 7a. To maximize the effectiveness of the FES test, extensive pre-experimental testing was conducted to determine the optimal electrode placement and stimulation voltage. Ultimately, the most effective configuration involved placing two electrodes on the forearm’s extensor digitorum muscle—approximately 8 cm from the wrist and 8 cm from the elbow—and applying a stimulation voltage of 15 V. Under this stimulation, the hand achieved significant extension with notably enhanced tension. The experiment was divided into two main phases: In the first phase, the exoskeleton remained in an unlocked state, and FES was alternately activated and deactivated to validate its effectiveness. In the second phase, with the exoskeleton in a locked state, FES was again toggled to assess the exoskeleton’s locking performance and its anti-fall capability. During the entire procedure, flex sensors embedded in the soft exoskeleton glove continuously recorded finger joint angles in real time to analyze hand motion under different conditions.

The experimental results are presented in Figure 7b and the Supplementary Video. From stage i to ii, with the exoskeleton unlocked and FES deactivated, the participant was able to freely open and close their hand, indicating that the system did not impede natural hand movements. In stage iii, although the exoskeleton remained unlocked, FES was activated. At this point, a significant decrease in finger flexion angles was observed, demonstrating that FES successfully triggered the contraction of the extensor digitorum, resulting in a non-voluntary, large-range hand extension. This confirmed the efficacy of the FES system. In stage iv, following the deactivation of FES, the participant was instructed to actively grasp a foot peg. The system was then triggered to lock and maintain the grasp posture. Once the exoskeleton was locked, the participant was instructed to relax their hand, relying solely on the locking force of the actuator to sustain the grip. This process verified the exoskeleton’s capability to maintain hand grasp independently without external force input. In stage v, while the exoskeleton remained locked, FES was reactivated, causing the extensor digitorum to contract and open the hand. However, due to the counteracting locking force of the exoskeleton, only the index and middle fingers extended slightly and maintained a semi-fist posture. This indicated that the exoskeleton system could effectively preserve the grasp when the user experienced involuntary hand extension. Meanwhile, the ring and little fingers, which were not constrained by the exoskeleton, were able to extend freely. Finally, in stage vi, with the exoskeleton still locked and FES deactivated, the hand returned to a fist posture, firmly gripping the foot peg. The results demonstrate that the locked exoskeleton effectively maintains grasp posture and resists involuntary hand opening, ensuring grip stability under fatigue or inattention.

### 3.2. Climbing Experiments

**Figure 8:**
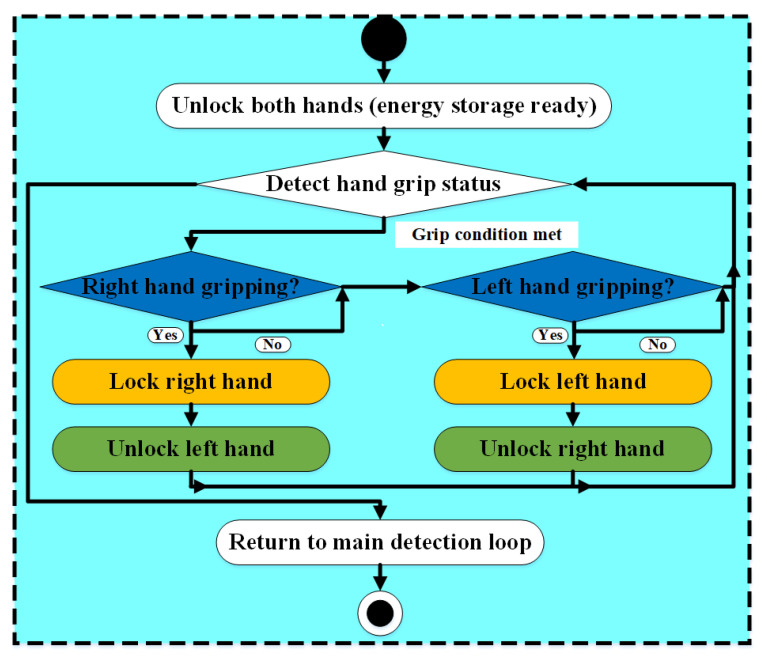
System workflow diagram for climbing test.

To validate the overall effectiveness of the soft exoskeleton system for fall prevention assistance during tower climbing, a simulated climbing experiment was conducted. The system assumes that the feet remain stably positioned on the climbing structure. Under this assumption, the control workflow (Figure 8) ensures that one hand is always locked while the other remains free to move, thereby maintaining a stable three- or four-point contact posture throughout the climbing process and enhancing safety [52]. Grip state recognition, which is critical for enabling timely locking, is determined by a sensor voltage threshold calculated from a weighted combination of all sensing signals. This threshold was empirically obtained through repeated grasping trials on the foot peg by a single participant, with weighting factors manually tuned to reflect the relative contributions of each sensing modality. Although effective in practice, this approach would benefit from further refinement and validation across a broader range of users. The experiment was conducted on a climbing machine inclined at an angle of 72.35°, closely approximating the actual climbing angle of tower workers. Prior to the experiment, the participant underwent safety training for the climbing machine, and the entire process was recorded from both front and rear views.

**Figure 9:**
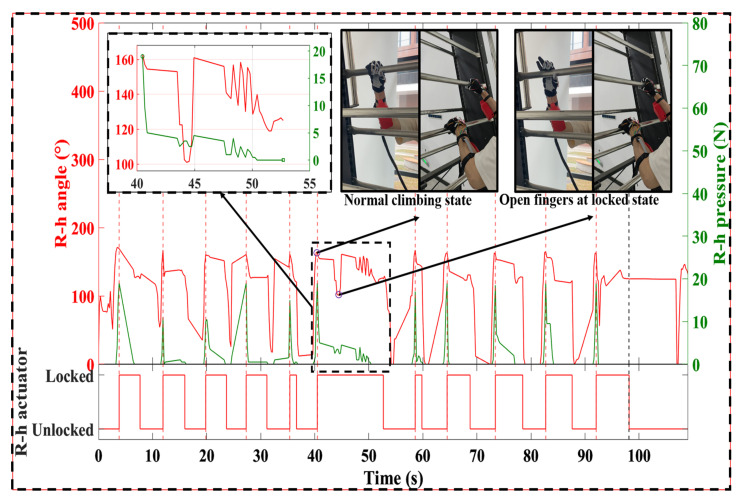
Climbing test results. R-h angle: right-hand flexion angle and R-h actuator: right-hand actuator states. R-h pressure: right-hand pressure.

After powering on, the system entered an initialization state in which the exoskeletons on both hands remained unlocked, allowing free movement. Once the participant’s right hand grasped the climbing bar, the right-hand exoskeleton automatically locked the grasping posture. When the left hand grasped the bar, the left-hand exoskeleton locked while the right-hand exoskeleton unlocked, ensuring that at least one hand remained in a locked state throughout the climbing process. During the experiment, the participant completed five cycles of alternating left- and right-hand climbing movements. At about 40 s, while the right hand was locked, the participant was instructed to voluntarily open it. The results showed that the participants were indeed able to actively open their hands to a certain extent, but the range of finger extension was limited by the locking mechanism. The experimental procedure and results are shown in Figure 9 and the Supplementary Video. For clarity, thumb flexion data—showing minimal variation during climbing—was excluded, and the average flexion angle of the index and middle fingers was used for assessment. Although the average flexion angle of the right-hand fingers decreased from 163° to 101° under strong voluntary extension, the hand still maintained a semi-grasp posture, demonstrating the effectiveness of the locking mechanism. At the end of the experiment (approximately 97 s), the participant pressed the reset button located at the waist, unlocking both exoskeletons and restoring free hand movement. This manual override ensures users can promptly disengage the system when necessary. This experiment demonstrates that the soft exoskeleton system can effectively maintain grasp posture and resist voluntary hand extension, ensuring hand stability during climbing.

## 4. Discussion

The proposed soft exoskeleton integrates a bioinspired rapid response actuator based on a spring energy storage–release strategy and a bionic partial finger constraint structure, with the objective of helping the climb of the tower and providing active fall prevention capability. Compared with conventional exoskeletons focused on load redistribution or fatigue mitigation, this system exhibits a distinct emphasis on rapid mechanical response and safety intervention. Its 37.5 ms locking time enables actuation faster than the typical human reflex latency of 80–120 ms [53], which enables timely provision of active prevention during tower climbing when workers’ hands relax or slip. This real-time intervention directly targets the key risk of falls in high-altitude climbing.

From a mechanical perspective, the PTFE-guided tendon pathway effectively balances friction reduction and efficient force transmission. The fixation points set at the phalangeal joints can form a favorable force lever, enabling each finger to generate sufficient torque while also ensuring the comfort of the user. The partial-finger configuration—constraining only the index and middle fingers—is based on ergonomic analyses showing these two digits contribute more than 70% of total grasping torque in power grasps [54,55]. This design preserves tactile sensation and ventilation for the remaining fingers, which is essential for long-duration operations under variable environmental conditions.

In a comparative context, recent works including Li et al. [32] and Mai et al. [49] have emphasized energy efficiency and structural simplicity. The current system complements these developments by focusing on millisecond-level response and mechanical fail-safe locking—a function rarely addressed in prior designs for occupational safety. Moreover, the adaptive grip-state recognition based on empirical thresholds shows promising robustnes but remains user-specific. The future integration of machine-learning-based adaptive calibration, inspired by the adaptive strategies from Park et al. [39], could enhance the system’s generalizability across users with diverse hand sizes and grip habits.

While the present results confirm the system’s functional feasibility, several limitations remain. The current validation was conducted on a single healthy subject under controlled conditions, which may not fully represent field scenarios such as professional tower climbing or involuntary hand motions induced by fatigue or reflexes. Moreover, the partial-finger actuation design, though enhancing dexterity, provides a relatively limited overall locking force compared to full-hand systems. These aspects will be addressed in future studies to further evaluate long-term reliability and ergonomic adaptability.

## 5. Conclusions

This work presents a soft exoskeleton designed to enhance hand grip and provide active fall prevention assistance during tower climbing. The system achieves a 37.5 ms mechanical locking response, a 0.72 kg lightweight profile, and 0.34 N·m torque output per finger, effectively combining portability with safety responsiveness. The integration of PTFE low-friction tendon guidance and partial-finger locking architecture offers a balanced trade-off between comfort, mobility, and secure grasping. This study confirms that millisecond-scale mechanical intervention can operate faster than human reflexes, indicating its feasibility for active fall mitigation in high-risk manual operations. Future work will expand subject recruitment to include professional electrical maintenance personnel, with evaluations focusing on long-term ergonomics, muscle fatigue, and behavioral adaptation. Testing will be extended to additional scenarios such as fatigue conditions. Durability assessments will be intensified to better characterize overall system performance. Machine learning algorithms, such as SVM, decision tree, and gradient-boosting methods, will be employed to adapt the grip recognition threshold for improved reliability across users.

## Data Availability

The original contributions presented in this study are included in the article. Further inquiries can be directed to the corresponding author(s).

## Supplementary Materials

The following supporting information can be downloaded at https://www.mdpi.com/article/10.3390/biomimetics10110721/s1, Supplementary videos for FES experiment and climbing experiment.
